# Vitamin B12 Deficiency Disguised As Hemolytic Anemia: A Case Presentation

**DOI:** 10.7759/cureus.51815

**Published:** 2024-01-07

**Authors:** Thaer A Abdul Hadi, Snehaja Ananthasivan, Aline Bitarelli, Erlyn Smith

**Affiliations:** 1 Pediatrics, University of Florida, Ascension Sacred Heart, Pensacola, USA; 2 Pediatric Hematology and Oncology, University of Florida, Ascension Sacred Heart, Pensacola, USA

**Keywords:** vitamin b12-induced hemolytic anemia, anemia, nutritional deficiency, vitamin b12 deficiency anemia, cobalamin deficiency

## Abstract

This case report describes an 18-month-old male presenting with hemolytic anemia and lethargy, who was ultimately diagnosed with severe vitamin B12 deficiency. The child exhibited global developmental delays, including a lack of speech and walking skills. Initially suspected as intravascular hemolytic anemia, the normal reticulocyte count led to further investigation, which revealed low cobalamin (vitamin B12) levels. The patient received vitamin B12 injections, resulting in normalized cobalamin levels. Additional evaluations ruled out metabolic disorders and other etiologies for his anemia. Follow-up laboratory testing showed improved hemoglobin levels, and the patient was discharged with plans for close monitoring. The case emphasizes the importance of considering vitamin B12 deficiency in children, particularly those with developmental delays and anemia. Early diagnosis and treatment are crucial for preventing long-term neurological consequences associated with severe and prolonged cobalamin deficiency. Healthcare professionals should be aware of the impact of nutrient deficiencies on growth, development, and brain maturation in pediatric populations.

## Introduction

Vitamin B12 deficiency in children is rare but treatable. It is more common in developing countries, and in developed countries, it usually occurs in exclusively breastfed infants. Symptoms are unspecific, such as failure to thrive, developmental delay, and lethargy. Early recognition is important to avoid permanent neurologic damage and will dictate long-term prognosis. Treatment with intramuscular cobalamin injections corrects the abnormalities, with improvement in activity seen within 24 to 48 hours, but neurodevelopmental recovery may not be complete. We present a case of vitamin B12 deficiency that caused significant developmental delay, lethargy, and hemolysis. The objective of this study is to describe the presentation of an 18-month-old male with hemolytic anemia caused by vitamin B12 deficiency and discuss the pathophysiology. 

## Case presentation

An 18-month-old male was sent to our pediatric emergency department from his pediatrician's office for anemia with lethargy. The mother was concerned about lethargy and decreased activity level of one-month duration that significantly worsened in the last few days.

Upon interviewing the mother, she admitted that he had been mainly breastfeeding for his nutrition since birth and rarely took smooth or liquid food. He very frequently gags and spits up solid food when introduced to him. Mom also reported constipation recently but denied any bloody or dark stools or any other sources of bleeding. He did not have any recent fevers or illnesses.

He was found to have global developmental delays on our evaluation, as the mother indicated that he still had not developed any speaking skills or started walking, which are normally expected for his age. He has not been evaluated for developmental delays or feeding difficulties in the past and was never on any supplements or medications. He has no known prior medical conditions.

Upon the clinical evaluation in the emergency department, he was noticed to have obvious pallor and lethargy and showed apathy during the physical exam. His vital signs were appropriate for his age, including his heart rate. He had no jaundice, scleral icterus, hepatomegaly, splenomegaly, or any abdominal masses.

His laboratory workup confirmed a low hemoglobin level of 8 g/dL with a hematocrit of 22.5%. MCV was 104 fL. RDW was elevated at 27.7%. WBC and platelet count were normal. Peripheral smear showed normochromic macrocytic anemia with occasional hypersegmented neutrophils.

Metabolic labs showed slightly elevated liver markers with an AST of 167 IU/L, an ALT of 98 IU/L, and a total bilirubin level of 2.4 mg/dL with 0.6 mg/dL of direct bilirubin. His initial CBC and metabolic panel were followed by labs to screen for hemolysis. LDH was markedly elevated at 3325 IU/L, but the corrected reticulocyte count was inappropriately normal at 1.2%. Haptoglobin was low (<8 mg/dL). Lead toxicity was excluded by a low level of 3.3 mcg/dL.

He was initially concluded to have ongoing hemolysis, but his normal reticulocyte count confused the diagnosis in the beginning.

Considering his macrocytic anemia, folate deficiency, vitamin B12 deficiency, and hypothyroidism were considered. His folate level was normal, and TSH and T4 were appropriate, but his cobalamin level resulted low at 146 pg/ml with elevated homocysteine (105 mcmol/L) and MMA (114 mcmol/L) levels, concluding that his anemia was, in fact, a result of cobalamin deficiency (Table 1).

**Table 1:** Laboratory results at the time of hospital admission

Lab (Units)	Lab Value	Normal Value
Hemoglobin (g/dL)	8	10.5 - 13.5
Hematocrit (%)	22.5	33 - 39
WBC count (K/uL)	9.4	6 - 17
Platelet count (K/uL)	263	150 - 350
MCV (fL)	104	70 - 86
RDW (%)	27.7	12 - 15
Corrected reticulocyte count (%)	1.2	0.5 – 2.5
AST (IU/L)	167	9 - 80
ALT (IU/L)	98	5 - 45
Total bilirubin (mg/dL)	2.4	<1.2
Direct bilirubin (mg/dL)	0.6	<0.2
LDH (IU/L)	3325	155 - 345
Haptoglobin (mg/dL)	<8	26 - 185
Cobalamin level (pg/ml)	146	260 - 1200
Folate level (ng/ml)	14.2	4 - 20
Lead level (mcg/dL)	3.3	<5
Homocysteine (mcmol/L)	105	4 - 15
Methylmalonic acid (mcmol/L)	114	0.04 – 0.4

Metabolic disorders were excluded by normal serum amino acids and normal organic acid testing. The patient received five days of vitamin B12 IM injections as recommended by the pediatric hematology-oncology team. Cobalamin levels normalized following the injections.

A workup was done to evaluate his oral aversion. He underwent upper GI studies and neck soft tissue imaging, which were normal. EGD showed evidence of gastritis and was further evaluated for autoimmune gastritis, considering his low B12 levels. Anti-parietal cells and gastric cell antibodies were both negative. Biopsy results were normal as well. The celiac panel was negative.

The patient was also evaluated by the speech therapy team and was found to have a significant oral aversion, for which he received therapy. He improved significantly and was able to tolerate soft and hard food for the first time.

Repeat blood testing on day nine of admission showed a hemoglobin level of 12.5 g/dL with an MCV of 97.7 fL. The patient was discharged within ten days with plans for close follow-ups with his pediatrician and the pediatric hematology team.

## Discussion

Vitamin B12 deficiency usually results from inadequate consumption of cobalamin-rich foods or difficulty absorbing it caused by the lack of intrinsic factors as in pernicious anemia or following gastric surgeries [1]. It is known that humans cannot synthesize cobalamin and are fully dependent on dietary sources, with the body stores in adults usually lasting for several years after restriction of intake or malabsorption [2].

Vitamin B12 deficiency in the pediatric population is mostly related to insufficient dietary intake. During infancy, cobalamin deficiency is a result of low maternal cobalamin stores during pregnancy, resulting in low liver stores in the infant, and maternal deficiencies after delivery will result in low contents of cobalamin in breast milk leading to low supplies to the already deficient infant who might become symptomatic as soon as two to 12 month of age [3,4]. Consequently, it is more common in exclusively breastfed infants by mothers with a strict vegetarian diet or who do not have access to food of animal origin for socio-economic reasons and is commonly described in India and Pakistan [5,6]. The patient that we describe likely had cobalamin deficiency from a lack of any animal foods in his diet and was solely dependent on breast milk for his nutrition. Although the mother denied any limitation as to her diet before and during pregnancy and her adherence to multivitamins during pregnancy, his cobalamin deficiency might be related to low maternal stores during pregnancy and while breastfeeding.

Cobalamin has a critical role in bone marrow maturation, nervous system development, and myelination. It is essential during infancy and childhood for normal and appropriate development, considering the very active myelination during those periods [7]. When deficient, it will interfere with myelination and may severely affect the CNS, resulting in brain atrophy [8].

Symptoms of severe B12 deficiency in infancy include lethargy, apathy with little interest in their surroundings, failure to thrive, convulsions, and developmental delays, which were consistent with the clinical picture of our patient at the time of presentation as he had global developmental delays with lethargy and hypoactivity that persisted even after his hemoglobin normalized [5].

Neurodevelopmental recovery may not be complete even after B12 stores are replenished, resulting in long-term cognitive and language deficits, especially when diagnosis and treatment are delayed [8,9]. Unfortunately, the patient was lost to follow-up after discharge, and long-term outcomes could not be assessed.

As noticed on his laboratory results, he had macrocytic anemia with hemolysis suggested by elevated indirect bilirubin, severely elevated LDH, and low haptoglobin but with a normal reticulocyte count.

Macrocytic and megaloblastic changes are caused by decreased nucleic acid metabolism with decreased cell division and normal cytoplasmic maturation, leading to nuclear-cytoplasmic desynchrony [10]. Severe deficiencies can inhibit the maturation of all bone marrow cell lines, leading to anemia, thrombocytopenia, and leukopenia, but our patient had normal white blood cell and platelet counts [11].

In a study including 201 adults with well-documented cobalamin deficiency, pancytopenia, leukopenia, and thrombopenia were described. Hemolytic anemia was present in 1.5% of the included population [11].

The exact process of hemolysis in patients with megaloblastic anemia is not fully understood with many theories listed in the literature, but it is believed to be mainly caused by the destruction of abnormal, immature, nucleated erythrocytes in the bone marrow, which is known as ineffective erythropoiesis [12].

It is also thought that increased levels of homocysteine with B12/folate deficiency can lead to oxidative stress damaging RBC membrane lipids and lead to intra and extramedullary hemolysis and that large RBCs passing through narrow capillaries will lead to shear forces with extramedullary hemolysis [13-15].

Hemolysis due to cobalamin deficiency can be confused with processes related to microangiopathic hemolysis, such as thrombocytopenic purpura (TTP) and HUS, considering that it can present as hemolytic anemia with low platelets, and thus it is sometimes referred to as pseudo thrombotic microangiopathy (TMA). In a study comparing patients with pseudo-TMA due to vitamin B12 deficiency with TTP, Noel et al. found the reticulocyte count to be low in pseudo-TMA cases in contrast to patients with TTP and concluded that very high LDH and a low reticulocyte count strongly suggest pseudo-TMA and cobalamin deficiency instead of a true thrombotic process with hemolysis [16]. Identifying hyper-segmented neutrophils and low cobalamin levels with an elevation of methylmalonic acid and or homocysteine is key in establishing B12 deficiency as a cause of hemolytic anemia [17].

B12 supplementation in pregnant and lactating women and the greater use of complementary B12-rich foods in infants after six months of age are suggested strategies to prevent deficiencies in infants [18].

Hematological findings improve soon after starting treatment with normalization of anemia within eight weeks. Neurological manifestation may show improvement as soon as one week after starting treatment and may take up to a few months to reach full recovery, but as discussed before, long-term irreversible neurological limitations are feared outcomes of severe and prolonged untreated vitamin B12 deficiency [19].

## Conclusions

In this article, we describe a toddler presenting with hemolytic anemia and developmental delays that were caused by severe vitamin B12 deficiency. Although vitamin B12 deficiency anemia is well recognized in children in certain parts of the world due to cultural and socio-economic reasons, it remains rare and unusual in many other countries, and it manifesting as hemolytic anemia can lead to delays in making an accurate diagnosis. We are highlighting the importance of being familiar with such medical diagnoses in pediatric patients, considering how critical it is to make an early diagnosis and initiate management and knowing the impact of such deficiencies on different aspects of growth, development, and brain maturation in such age groups.
